# The Mediating Role of Body Mass Index in the Association of Socioeconomic Status With Hepatic Steatosis and Liver Fibrosis: A Cross-Sectional Study Based on NHANES 2021–2023

**DOI:** 10.1155/ije/4478977

**Published:** 2025-09-09

**Authors:** Zongnan Chen, Xiaoling Zhu, Juan Guo, Gang Ma

**Affiliations:** ^1^Postdoctoral Research Station, Agricultural Bank of China, Beijing, China; ^2^China School of Banking and Finance, University of International Business and Economics, Beijing, China; ^3^Department of Nursing, Dazhou Central Hospital, Dazhou, Sichuan, China; ^4^Department of Gastroenterology, The First People's Hospital of Guangyuan, Guangyuan, Sichuan, China; ^5^Department of Hepatobiliary Surgery, The First People's Hospital of Guangyuan, Guangyuan, Sichuan, China

**Keywords:** body mass index, hepatic steatosis, liver fibrosis, mediation, NHANES, socioeconomic status

## Abstract

**Background:** Socioeconomic status (SES) influences a wide range of health outcomes, including hepatic steatosis and liver fibrosis, which are increasingly concerning. The aim of the study was to investigate the association between SES and hepatic steatosis and liver fibrosis and examine the potential mediating effects of body mass index (BMI) in this association.

**Methods:** We used the National Health and Nutrition Examination Survey (NHANES) 2021–2023 data to conduct a cross-sectional study. Occupation, insurance, family income level, and education level were employed as indicators of SES. Hepatic steatosis and liver fibrosis were quantified by controlled attenuation parameter (CAP) and liver stiffness measurement (LSM), respectively. Mediation analysis was used to estimate the direct and indirect associations of SES with hepatic steatosis and liver fibrosis through BMI after adjustment for potential confounders.

**Results:** The study included 4455 participants. Compared to individuals with low SES, those with high SES had a lower risk of hepatic steatosis (odds ratios [OR] = 0.80, 95% CI: 0.69–0.94, *p* < 0.01) and liver fibrosis (OR = 0.77, 95% CI: 0.61–0.97, *p*=0.03). However, after adjusting for confounding factors, the associations were no longer statistically significant (hepatic steatosis: OR = 0.90, 95% CI: 0.75–1.08, *p*=0.25; liver fibrosis: OR = 0.87, 95% CI: 0.67–1.15, *p*=0.32). BMI differed significantly across SES grades (*p*=0.04). Restricted cubic spline analysis revealed a significant nonlinear positive association between BMI and hepatic steatosis (*p* < 0.01), and a linear positive association with liver fibrosis (*p*=0.11). Moreover, BMI accounted for 32.8% of the mediation effect between SES and hepatic steatosis and 18.2% of the mediation effect between SES and liver fibrosis.

**Conclusion:** People with higher SES are less likely to develop hepatic steatosis and liver fibrosis, although the associations were attenuated after adjustment for confounding factors. SES might contribute to hepatic steatosis and liver fibrosis through the involvement of BMI.

## 1. Introduction

Nonalcoholic fatty liver disease (NAFLD) is one of the most prevalent chronic liver diseases globally and one of the primary causes of advanced liver disease, with a global prevalence estimated at around 25% [1]. In the United States alone, over 80 million people are diagnosed with NAFLD, and in Asia, it exceeds 30%. With diet and lifestyle modifications, the prevalence of NAFLD is estimated to rise to reach 50% of the world population by the year 2040 [2, 3]. The pathology starts with noncomplex hepatic steatosis, progressing to nonalcoholic steatohepatitis (NASH), liver fibrosis, cirrhosis, and finally hepatocellular carcinoma [4]. Vibration-controlled transient elastography (VCTE) is usually used for the screening of NAFLD, as it is not invasive and has satisfactory accuracy. It measures the controlled attenuation parameter (CAP) and liver stiffness measurement (LSM), which are used to assess the severity of hepatic steatosis and fibrosis, respectively [5].

Socioeconomic status (SES), such as occupation, insurance, family income level, and level of education, is a significant predictor of access to healthcare, diet, and lifestyle, which can subsequently affect health [6, 7]. Individuals of lower SES have a higher risk of obesity, dietary insufficiencies, and limited access to preventive healthcare services and thus may be at a higher risk for liver disease [8, 9]. Studies have reported a negative association between income status of the family and incidence of metabolic syndrome [10]. Underinsured or uninsured patients are bound to be diagnosed late when liver disease becomes severe [11]. Additionally, patients with higher education levels will have better health literacy, healthier lifestyles, and increased use of medical care. Lower educational attainment is associated with increased NAFLD rates, progression of liver fibrosis, and death from liver disease [12, 13].

Increased body mass index (BMI) promotes fat tissue deposition, particularly an increase in visceral fat, which triggers hepatic fat accumulation and eventually leads to hepatic steatosis. It also triggers inflammatory pathways and oxidative stress, which ultimately lead to cell damage in the liver and further progression of liver fibrosis [14]. There have been numerous studies showing that patients with higher BMI are likely to develop liver fibrosis and hepatic steatosis [15, 16]. As the connection between SES and health outcomes is multifaceted and influenced by lifestyle, the role of BMI in the SES-health outcomes relationship is critical to recognize in designing interventions aimed at reducing health disparities and improving liver health.

The aim of this research is to explore the relationship between SES, hepatic steatosis, and liver fibrosis and the mediating role of BMI based on National Health and Nutrition Examination Survey (NHANES) 2021–2023 data. Through this analysis, we aim to gain a deeper insight into the social determinants of liver disease and highlight BMI as a modifiable factor in these associations.

## 2. Methods

### 2.1. Study Design and Population

This study utilized publicly available data from NHANES, a national surveillance system that assesses the nutritional and health status of US residents. The National Center for Health Statistics (NCHS) conducts the survey every 2 years, collecting data using specifically designed mobile examination centers (MEC) and household interviews. The NCHS Research Ethics Review Board approved the NHANES program, and informed written consent was provided by all participants (the website was https://www.cdc.gov/nchs/nhanes/about/erb.html).

**Figure 1:**
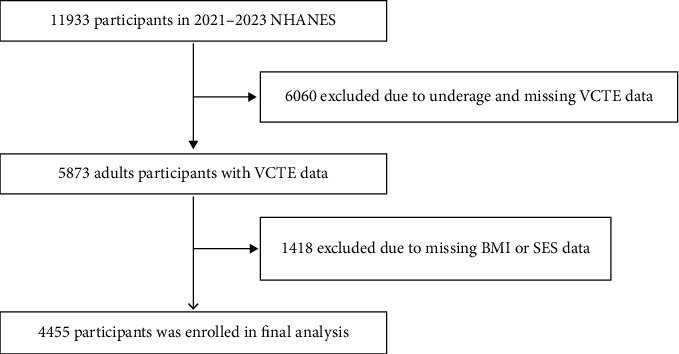
Flowchart of subject inclusion and exclusion.

Our study collected data from 2021 to 2023, and a total of 11,933 participants were included. Minors and those without elastography tests were excluded, and 5873 participants remained. Subsequently, participants with incomplete BMI and SES data were excluded, resulting in a final analytic sample of 4455 individuals (Figure 1). Detailed study information is available at https://wwwn.cdc.gov/nchs/nhanes/continuousnhanes/default.aspx?Cycle=2021-2023.

### 2.2. Data Collection

We collected comprehensive participant data across four domains:

• Demographics: age, gender, and race
• SES indicators: occupation, family income level, health insurance, and education level
• Clinical measurements: BMI, CAP, and LSM
• Medical history: hypertension, hypercholesterolemia, diabetes, smoking (defined as having smoked at least 100 cigarettes in life), and alcohol consumption (any kind of alcohol).
• Laboratory parameters: low-density lipoprotein (LDL), high-density lipoprotein (HDL), C-reactive protein (CRP), and glycohemoglobin.

### 2.3. SES Assessment

The SES of participants was divided into three grades based on factors such as occupation, household income level, medical insurance, and education level: high SES, medium SES, and low SES (Table S1). This multidimensional approach has been employed in several studies and has been shown to effectively reflect the impact of SES on health [17–20]. Occupation was divided into employment and unemployment; Family income level was calculated using the family income to poverty ratio (FIPR) and was divided into three groups: ≥ 3.5, ≥ 1.3 to < 3.5, and < 1.3 [21, 22]; health insurance status includes private, public, or uninsured; the education level was divided into university or above, high school, and below high school.

### 2.4. Definition of Hepatic Steatosis and Liver Fibrosis

Hepatic steatosis was defined by a median CAP ≥ 285 dB/m, a threshold that has shown high sensitivity in studies and effectively identifies hepatic fat [23, 24]. Liver fibrosis was defined by a LSM ≥ 8.6 kPa, a standard that demonstrated good sensitivity and specificity in studies, effectively distinguishing between no fibrosis and fibrosis, and providing strong diagnostic value [21, 25].

### 2.5. Statistical Analysis

All statistical analyses were conducted with R 4.2.2. Baseline comparisons between participants of varying SES grade participants were conducted using *t*-tests and *χ*^2^ tests for continuous and categorical variables, respectively. Where appropriate, results are presented as means ± SDs for continuous variables and proportions for categorical variables. The Kruskal–Wallis test was used to ascertain the overall difference in BMI between the three SES groups (low, medium, and high). If the Kruskal–Wallis test was significant, Dunn's test was performed for pairwise comparisons with Holm's adjustment for multiple comparisons.

Logistic regression analysis was utilized to investigate the association between SES and liver fibrosis and hepatic steatosis. Allusion was made to the low SES group among middle and high SES groups. The results were presented in the form of odds ratios (OR) and the corresponding 95% confidence intervals (CI). In this model, Model 1 was not covariate-adjusted, and Model 2 was covariate-adjusted for sociodemographic factors, i.e., gender, age, and race. Model 3 also covariate-adjusted for those factors that could have a potential impact on the outcomes, i.e., smoking, alcohol consumption, hypertension, hyperlipidemia, and diabetes, in addition to Model 2 adjustments.

Mediation analysis was used to assess the potential mediating effects of BMI in the relationship between SES and hepatic steatosis and liver fibrosis. Analysis was carried out with the “mediate” from the mediation package in R, with a nonparametric bootstrap method (with 1000 resampling iterations) to estimate the indirect effect, direct effect, total effect, along with their 95% CI and *p* values. In addition, we used the Restricted Cubic Splines (RCS) method to examine the potential nonlinear relation between BMI and hepatic steatosis and liver fibrosis. A logistic regression model was built with the “lrm” of the rms package, and statistical significance of the overall and nonlinear component pieces was tested with the Wald *χ*^2^ test. We also conducted subgroup analysis, which included seven subgroups stratified by age, sex, race, alcohol use, smoking, history of hypertension, history of high cholesterol, and history of diabetes. In sensitivity analyses, we assessed the robustness of the results by replacing the comprehensive SES stratification with FIPR-based stratification and excluding missing values. Two-tailed *p* < 0.05 is statistically significant.

## 3. Results

### 3.1. Participant Characteristics

The baseline participant characteristics are contained in Table 1. On the demographic aspect, the high SES group had excellent representation in the form of a significantly large percentage of participants (58.5%) versus medium (20.9%) and low SES groups (20.5%). Distribution by gender was also evenly distributed across the SES groups with females dominating all the SES groups (53.8% among low SES, 57.4% among medium SES, and 54.4% among high SES). By race, the greatest proportion of participants was non-Hispanic Whites (61.4%). The medium SES group reported the highest mean BMI (30.3), and the high SES group reported the lowest mean BMI (20.4). Participants in the low SES group reported higher prevalence of hypertension and diabetes and were more likely to be unemployed, have lower education, and have lower incomes, with many reporting public or no health insurance. Also, the prevalence of alcohol use and smoking was high in the low SES group as well.

### 3.2. Correlations of SES With Hepatic Steatosis and Liver Fibrosis

The association of SES with hepatic steatosis and liver fibrosis is shown in Table 2. Logistic regression analysis showed an inverse correlation between SES and risk for hepatic steatosis and liver fibrosis. In Model 1, individuals with high SES had significantly lower chances of hepatic steatosis (OR = 0.80, 95% CI: 0.69–0.94, *p* < 0.01) and liver fibrosis (OR = 0.77, 95% CI: 0.61–0.97, *p*=0.03). This association persisted after adjusting for sex, age, and race in Model 2 (hepatic steatosis: OR = 0.81, 95% CI: 0.70–0.95, *p*=0.01; liver fibrosis: OR = 0.77, 95% CI: 0.62–0.98, *p*=0.03). However, after further control for smoking, alcohol consumption, hypertension, hyperlipidemia, and diabetes in Model 3, the risk association was diminished and no more significant (hepatic steatosis: OR = 0.90, 95% CI: 0.75–1.08, *p*=0.25; liver fibrosis: OR = 0.87, 95% CI: 0.67–1.15, *p*=0.32).

### 3.3. Subgroup Analysis and Sensitivity Analysis

**Figure 2:**
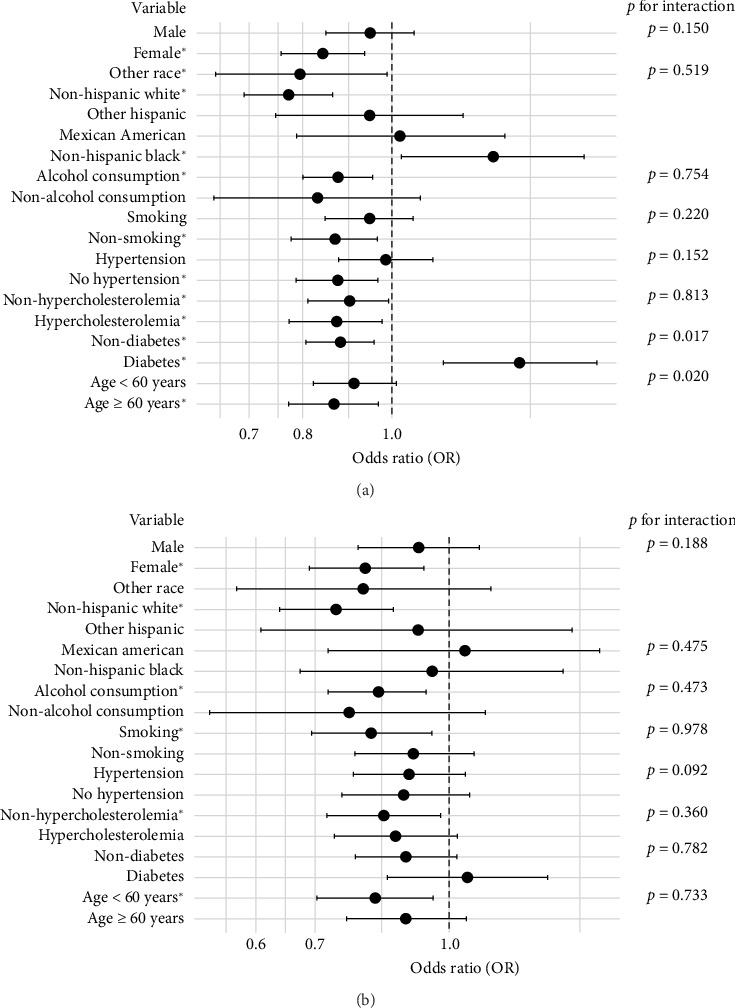
Subgroup analysis of socioeconomic status associated with hepatic steatosis and liver fibrosis. (a) Stratified analysis: SES and hepatic steatosis. (b) Stratified analysis: SES and liver fibrosis. ^∗^Indicates that the variable has statistical significance (*p* < 0.05).

Figure 2 shows the results of the subgroup analysis. It revealed that SES was inversely associated with both hepatic steatosis and liver fibrosis in subpopulations such as females, alcohol consumers, non-Hispanic Whites, and individuals without hypercholesterolemia (*p* < 0.05). The results also indicated that the association between SES and hepatic steatosis was stronger in individuals with diabetes and those under 60 years of age (*p* for interaction < 0.05), while the association between SES and liver fibrosis was not influenced by any factors.

We also conducted sensitivity analyses. Replacing comprehensive SES stratification with the FIPR indicator showed that the high FIPR group was associated with a lower risk of hepatic steatosis and liver fibrosis. However, these associations became nonsignificant after adjusting for confounders (Table S2). In the analysis excluding missing values, high SES was linked to a lower risk of liver disease, but these associations also became nonsignificant after adjusting for smoking, alcohol, hypertension, and other factors (Tables S3 and S4). These analyses confirmed the robustness of our findings.

### 3.4. Correlations of BMI With Hepatic Steatosis and Liver Fibrosis

**Figure 3:**
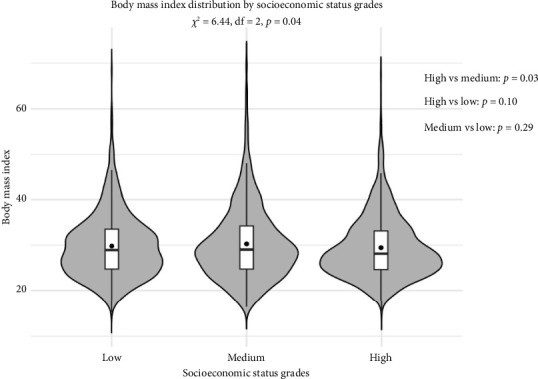
Distribution of body mass index across socioeconomic status grades.

**Figure 4:**
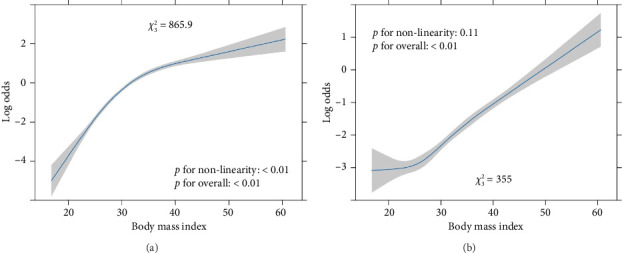
Restricted cubic spline curve of the relationship between body mass index and the risk of hepatic steatosis and liver fibrosis. (a) Hepatic steatosis. (b) Liver fibrosis.

Figure 3 presents the distribution of BMI across different SES levels. The results showed that there was a statistically significant difference in the overall BMI between the SES groups (*p*=0.04). The difference between the BMI of the high SES and medium SES groups was statistically significant (*p*=0.03), whereas those between the high and low SES groups (*p*=0.10), and between the low and medium SES groups (*p*=0.29), were not statistically significant. RCS analysis revealed a robust nonlinear positive relationship between BMI and hepatic steatosis (*p* < 0.01) (Figure 4(a)). By contrast, the association between BMI and liver fibrosis was linear or weakly nonlinear at best (*p*=0.11) (Figure 4(b)).

### 3.5. The Mediating Role of BMI on SES and Hepatic Steatosis and Liver Fibrosis

Table 3 shows the result of mediation analysis. The total mediating role of SES on hepatic steatosis (−0.027, 95% CI: −0.045 to −0.001, *p* < 0.001) and liver fibrosis (−0.019, 95% CI: −0.038 to −0.000, *p*=0.006) were significant through BMI. BMI accounted for 32.8% of the mediation effect between SES and hepatic steatosis, and 18.2% of the mediation effect between SES and liver fibrosis.

## 4. Discussion

Our analysis identified that high SES participants had significantly lower risks of liver fibrosis and hepatic steatosis. The associations, however, became weaker after adjustment for smoking, alcohol consumption, hypertension, hyperlipidemia, and diabetes. Subgroup analyses further indicated that inverse SES-risk associations for liver disease were more pronounced in certain groups, such as females, non-Hispanic Whites, and drinkers. Furthermore, BMI significantly differed according to SES and had a nonlinear association with hepatic steatosis but a linear or weakly nonlinear association with liver fibrosis. Mediation analysis indicated that BMI was involved in the association between SES and liver fibrosis, as well as hepatic steatosis.

Findings of the study concurred with current studies, which found that there was an inverse correlation between SES and risk of liver disease [26–28]. The relationship can be attributed to numerous factors including occupation, education, and lifestyle habits. For instance, an Italian multicenter study reaffirmed that poor lifestyle habits were major determinants for the development and worsening of NAFLD [29]. In another large sample study involving more than 10,000 participants, lower educational status independently increased the risk of NAFLD in Austrian adults [30]. Talens et al. further observed that working conditions, food insecurity, low health, and other determinant insurance coverage are linked with an increase in the global incidence rate of NAFLD and complications [31]. Surprisingly, our study found that associations between SES and liver disease risk were weakened following further adjustment for hypertension, hyperlipidemia, diabetes, smoking, and alcohol use. This finding not only confirmed that SES impacted the risk of liver disease indirectly through health-related behaviors but also highlighted the central role of clinical metabolic conditions—hypertension, hyperlipidemia, and diabetes—within this relationship. Low SES individuals often faced structural barriers, such as food deserts, unsafe housing conditions, and limited access to preventive healthcare services [32, 33]. These adverse conditions heightened the risk of obesity, insulin resistance, and chronic inflammation—the key factors involved in the pathogenesis of NAFLD and liver fibrosis [34].

Subgroup analysis also revealed that the reversed association between SES and liver disease was more pronounced in certain populations. Women with higher SES had a reduced risk of liver disease, which was accounted for by healthier lifestyles, better access to healthcare, and the protective effect of estrogen [35]. Individuals with high SES were also more likely to be moderate drinkers, typically preferring wine, which served to reduce the risk of alcohol-related illness [36]. The prevalence of the PNPLA3 risk allele was lower in non-Hispanic White subjects, which may have helped to account for their comparatively lower risk of NAFLD and liver fibrosis [37]. We also determined that BMI played a role in the association between SES and liver disease, suggesting that obesity may be an important biological pathway linking structural socioeconomic disadvantage to adverse liver outcomes.

Individuals with higher SES were likely to enjoy advantages in health literacy, diet quality, and access to physical activity opportunities, which enabled them to achieve a lower BMI compared to the middle and low SES groups [38, 39]. In America, metabolic dysfunction-associated steatotic liver disease (MASLD) was identified as higher in cases of low-income adolescents, particularly among Hispanic males with hypertension and obesity. Furthermore, for each 1-unit increase in BMI, there was an association with a 25% increase in the incidence of MASLD in this population [12]. Another study confirmed that patients who resided in areas with high Social Deprivation Index (SDI) were more likely to develop liver disease. Furthermore, when BMI was greater than 23 kg/m^2^, risk of fatty liver disease dramatically increased in a nonlinear fashion with every increment of 1 kg/m^2^ higher, again elevating the risk [40]. This is in agreement with our results and also supports the presence of a powerful nonlinear relationship between BMI and hepatic steatosis.

There are, however, some limitations to this research as well. First, the cross-sectional study design prevents the SES-liver disease outcome causality inference and restricts the demonstration of the temporal relationship between SES and NAFLD or liver fibrosis development. Second, residual confounding could still exist even after adjusting for multiple covariates due to uncontrolled factors such as dietary habits, exercise intensity, and sleep duration. Third, SES applies a multidimensional measurement framework. Though the methodology can be applied to measure a number of dimensions of the socioeconomic background, there might be inconsistency in the measures, and it might lead to classification bias. Finally, the current research utilized data from the NHANES, and the generalizability of the research results might be limited to other nations.

## 5. Conclusion

In the current study, the level of SES was negatively correlated with the risk of liver fibrosis and hepatic steatosis, and this correlation may be confounded by factors such as age, alcohol consumption, smoking, hypertension, hyperlipidemia, and diabetes. SES might contribute to hepatic steatosis and liver fibrosis through the involvement of BMI. Future large prospective cohort studies are required to further establish the correlations and identify the underlying pathways.

## Figures and Tables

**Table 1:** Characteristics of study participants.

Variables	Total sample	Low SES	Medium SES	High SES	*p* value
Number of participants, *n* (%)	4455	915 (20.5)	933 (20.9)	2607 (58.5)	
Age (years), *n* (%)					0.02
< 60	2437 (54.7)	524 (57.3)	475 (50.9)	1438 (55.2)	
≥ 60	2018 (45.3)	391 (42.7)	458 (49.1)	1169 (44.8)	
Gender, *n* (%)					0.19
Male	2010 (45.1)	423 (46.2)	397 (42.6)	1190 (45.6)	
Female	2445 (54.9)	492 (53.8)	536 (57.4)	1417 (54.4)	
Race, *n* (%)					2.03
Mexican American	278 (6.2)	134 (14.7)	44 (4.7)	100 (3.8)	
Other Hispanic	405 (9.2)	149 (16.3)	95 (10.2)	161 (6.2)	
Non-Hispanic white	2736 (61.4)	320 (35.0)	545 (58.4)	1871 (71.8)	
Non-Hispanic black	486 (10.9)	183 (20.0)	125 (13.4)	178 (6.8)	
Other race	550 (12.3)	129 (14.1)	124 (13.3)	297 (11.4)	
BMI, mean ± sd		29.8 ± 7.0	30.3 ± 8.0	29.4 ± 6.8	0.04
Insurance, *n* (%)					< 0.01
No insurance	348 (7.8)	273 (29.8)	35 (3.8)	40 (1.53)	
Public issuance	1656 (37.2)	556 (60.8)	715 (76.6)	385 (14.8)	
Private insurance	2451 (55.0)	86 (9.4)	183 (19.6)	2182 (83.7)	
Occupation, *n* (%)					2.70
Employment	2478 (55.6)	278 (30.4)	440 (47.2)	1760 (67.5)	
Unemployment	1977 (44.4)	637 (69.6)	493 (52.8)	847 (32.5)	
Education, *n* (%)					< 0.01
Less than high school	487 (10.9)	451 (49.3)	36 (3.8)	0 (0)	
High school or equivalent	2294 (51.5)	464 (50.7)	787 (84.4)	1043 (40.0)	
College or above	1674 (37.6)	0 (0)	110 (11.8)	1564 (60.0)	
FIPR, *n* (%)					< 0.01
< 1.3	1017 (22.8)	623 (68.1)	362 (38.8)	32 (1.23)	
≥ 1.3 to < 3.5	1842 (41.4)	292 (31.9)	431 (46.2)	1119 (42.9)	
≥ 3.5	1596 (35.8)	0 (0)	140 (15.0)	1456 (55.8)	
Alcohol consumption, *n* (%)					< 0.01
Yes	3616 (91.7)	615 (84.0)	728 (90.2)	2273 (94.6)	
No	326 (8.3)	117 (16.0)	79 (9.8)	130 (5.4)	
Smoking, *n* (%)					< 0.01
Yes	1892 (42.5)	507 (55.4)	446 (47.8)	939 (36.0)	
No	2563 (57.5)	408 (44.6)	487 (52.2)	1668 (64.0)	
Hypertension, *n* (%)					< 0.01
Yes	1660 (37.3)	389 (42.5)	386 (41.4)	885 (34.0)	
No	2795 (62.7)	526 (57.5)	547 (58.6)	1722 (66.0)	
Hypercholesterolemia, *n* (%)					0.29
Yes	1848 (41.3)	359 (39.2)	389 (41.7)	1100 (42.2)	
No	2617 (58.7)	556 (60.8)	544 (58.3)	1507 (57.8)	
Diabetes					< 0.01
Yes	585 (13.1)	190 (20.8)	141 (15.1)	254 (9.7)	
No	3870 (86.9)	725 (79.2)	792 (84.9)	2353 (90.3)	
CAP (dB/m), *n* (%)					< 0.01
≥ 285	1583 (35.5)	354 (38.7)	352 (37.7)	877 (33.6)	
< 285	2872 (64.5)	561 (61.3)	581 (62.3)	1730 (66.4)	
LSM (kPa), *n* (%)					< 0.01
≥ 8.6	515 (11.6)	118 (12.9)	131 (14.0)	266 (10.2)	
< 8.6	3940 (88.4)	797 (87.1)	802 (86.0)	2341 (89.8)	
LDL (mg/dL), mean ± sd		185.8 ± 45.8	185.4 ± 42.5	192.7 ± 40.8	< 0.01
HDL (mg/dL), mean ± sd		51.6 ± 13.7	54.1 ± 14.6	55.8 ± 15.0	< 0.01
Glycohemoglobin (%), mean ± sd		6.1 ± 4.5	5.8 ± 1.0	5.6 ± 0.9	< 0.01
CRP (mg/L), mean ± sd		4.2 ± 7.5	4.6 ± 9.0	3.3 ± 6.1	< 0.01

Abbreviations: BMI, body mass index; CAP, controlled attenuation parameter; CRP, C-reactive protein; FIPR, family income to poverty ratio; HDL, high-density lipoprotein; LDL, low-density lipoprotein; LSM, liver stiffness measurement; SES, socioeconomic status.

**Table 2:** Associations of socioeconomic status with hepatic steatosis and liver fibrosis.

	Model 1		Model 2		Model 3	
	OR (95% CI)	*p* value	OR (95% CI)	*p* value	OR (95% CI)	*p* value
*Hepatic steatosis*						
Low SES	Reference	—	Reference	—	Reference	—
Medium SES	0.96 (0.80–1.16)	0.67	0.98 (0.81–1.19)	0.86	0.96 (0.78–1.20)	0.74
High SES	0.80 (0.69–0.94)	< 0.01	0.81 (0.70–0.95)	0.01	0.90 (0.75–1.08)	0.25
*p* for trend	< 0.01		< 0.01		0.22	

*Liver fibrosis*						
Low SES	Reference	—	Reference	—	Reference	—
Medium SES	1.10 (0.85–1.44)	0.47	1.11 (0.85–1.45)	0.45	1.18 (0.87–1.60)	0.30
High SES	0.77 (0.61–0.97)	0.03	0.77 (0.62–0.98)	0.03	0.87 (0.67–1.15)	0.32
*p* for trend	< 0.01		< 0.01		0.14	

*Note:* Model 1: no confounding factors were adjusted. Model 2: adjusted for age, gender, race. Model 3: adjusted for age, gender, race, smoking, alcohol consumption, hypertension, hyperlipemia, diabetes.

Abbreviations: CI, confidence interval; OR, odds ratio; SES, socioeconomic status.

**Table 3:** The mediating effect of body mass index on the association of socioeconomic status with hepatic steatosis and liver fibrosis.

	Estimate	95% CI	*p* value
*Hepatic steatosis*			
Indirect effect	−0.009	−0.018 to 0.000	0.044
Direct effect	−0.018	−0.034 to 0.000	0.022
Total effect	−0.027	−0.045 to −0.001	< 0.001
Proportion mediated	32.8%	—	—

*Liver fibrosis*			
Indirect effect	−0.003	−0.007 to 0.000	0.044
Direct effect	−0.016	−0.033 to 0.000	0.018
Total effect	−0.019	−0.038 to 0.000	0.006
Proportion mediated	18.2%	—	—

Abbreviation: CI, confidence interval.

## Data Availability

The original contributions presented in the study are included in the article/Supporting Information; further inquiries can be directed to the corresponding authors.

## Supporting Information

Table S1. Definitions of low, medium, and high socioeconomic status.

Table S2. Associations of the family income to poverty ratio with hepatic steatosis and liver fibrosis.

Table S3. Missing of variables.

Table S4. The relationship between socioeconomic status and hepatic steatosis and liver fibrosis was evaluated after excluding data with missing values.
