# Supplementary material for: The Mediating Role of Body Mass Index in the Association of Socioeconomic Status With Hepatic Steatosis and Liver Fibrosis: A Cross-Sectional Study Based on NHANES 2021–2023
Source: Int J Endocrinol. 2025 Sep 9;2025:4478977. doi: 10.1155/ije/4478977 (PMC12440650; doi:10.1155/ije/4478977)
Supplement: Supporting Information — Additional supporting information can be found online in the Supporting Information section. [file 4478977.f1.docx]

**Supplementary file**

**Table S1. Definitions of low, medium, and high socioeconomic statu**

**Table S2. Associations of the family income to poverty ratio with hepatic steatosis and liver fibrosis**

**Table S3. Missing of variables**

**Table S4. The relationship between socioeconomic status and hepatic steatosis and liver fibrosis was evaluated after excluding data with missing values.**

**Table S1. Definitions of low, medium, and high socioeconomic status**

|  | | Employment | | | Unemployment | | |
| --- | --- | --- | --- | --- | --- | --- | --- |
|  |  | FIPR≥1.3 | FIPR≥1.3 to < 3.5 | FIPR<1.3 | FIPR≥3.5 | FIPR≥1.3 to < 3.5 | FIPR<1.3 |
| Private health insurance | College or above | High | Medium | Medium | High | Medium | Low |
|  | High school | High | Medium | Medium | High | Medium | Low |
|  | Below high school | High | Medium | Low | High | Medium | Low |
| Public health insurance | College or above | High | Medium | Low | High | Low | Low |
|  | High school | High | Medium | Low | Low | Low | Low |
|  | Below high school | Low | Low | Low | Low | Low | Low |
| No health insurance | College or above | High | Medium | Low | Low | Low | Low |
|  | High school | Medium | Medium | Low | Low | Low | Low |
|  | Below high school | Low | Low | Low | Low | Low | Low |
| FIPR: the ratio of family income to poverty. | | | | | | | |

**Table S2. Associations of the family income to poverty ratio with hepatic steatosis and liver fibrosis**

|  | **Model 1** | | **Model 2** | | **Model 3** | |
| --- | --- | --- | --- | --- | --- | --- |
|  | **OR (95% CI)** | **P-value** | **OR (95% CI)** | **P-value** | **OR (95% CI)** | **P-value** |
| **Hepatic steatosis** | | | | | | |
| Low FIPR | reference | - | reference | - | reference | - |
| Medium FIPR | 1.10 (0.94-1.29) | 0.25 | 1.02 (0.87-1.20) | 0.82 | 1.09 (0.91-1.31) | 0.35 |
| High FIPR | 0.90 (0.76-1.06) | 0.20 | 0.83 (0.70-0.99) | 0.03 | 0.91 (0.75-1.11) | 0.35 |
| P for trend | 0.11 | | 0.02 | | 0.20 | |
| **Liver fibrosis** | | | | | | |
| Low FIPR | reference | - | reference | - | reference | - |
| Medium FIPR | 1.07 (0.84-1.35) | 0.59 | 0.95 (075-1.21) | 0.68 | 1.11 (0.85-1.46) | 0.44 |
| High FIPR | 0.86 (0.67-1.11) | 0.24 | 0.77 (0.60-1.00) | 0.05 | 0.91 (0.68-1.23) | 0.55 |
| P for trend | 0.17 | | 0.04 | | 0.40 | |
| Abbreviations: FIPR: the family income to poverty ratio; OR: odds ratio; CI: confidence interval.  Low FIPR: < 1.3; Medium FIPR: ≥ 1.3 to < 3.5; High FIPR: ≥ 3.5.  Model 1: No confounding factors were adjusted.  Model 2: Adjusted for Age, Gender, Race.  Model 3: Adjusted for Age, Gender, Race, Smoking, alcohol consumption, hypertension, hyperlipemia, diabetes. | | | | | | |

**Table S3. Missing of variables**

| **Variable** | **Missing value** | **Missing proportion** |
| --- | --- | --- |
| Alcohol consumptio | 513 | 11.5% |
| Low density lipoprotein | 374 | 8.4% |
| High density lipoprotein | 374 | 8.4% |
| Glycohemoglobin | 182 | 4.1% |
| C-reactive protein | 304 | 6.8% |

**Table S4. The relationship between socioeconomic status and hepatic steatosis and liver fibrosis was evaluated after excluding data with missing values.**

|  | **Model 1** | | **Model 2** | | **Model 3** | |
| --- | --- | --- | --- | --- | --- | --- |
|  | **OR (95% CI)** | **P-value** | **OR (95% CI)** | **P-value** | **OR (95% CI)** | **P-value** |
| **Hepatic steatosis** | | | | | | |
| Low SES | reference | - | reference | - | reference | - |
| Medium SES | 0.84 (0.68-1.05) | 0.13 | 0.85 (0.68-1.06) | 0.15 | 0.91 (0.72-1.15) | 0.42 |
| High SES | 0.75 (0.63-0.90) | < 0.01 | 0.73 (0.60-0.88) | < 0.01 | 0.84 (0.68-1.03) | 0.09 |
| P for trend | < 0.01 | | < 0.01 | | 0.08 | |
| **Liver fibrosis** | | | | | | |
| Low SES | reference | - | reference | - | reference | - |
| Medium SES | 1.09 (0.80-1.50) | 0.58 | 1.05 (0.76-1.45) | 0.77 | 1.15 (0.82-1.62) | 0.41 |
| High SES | 0.75 (0.58-0.99) | 0.04 | 0.69 (0.52-0.93) | 0.01 | 0.80 (0.59-1.10) | 0.16 |
| P for trend | < 0.01 | | < 0.01 | | 0.04 | |
| Abbreviations: SES: socioeconomic status; OR: odds ratio; CI: confidence interval.  Model 1: No confounding factors were adjusted.  Model 2: Adjusted for Age, Gender, Race.  Model 3: Adjusted for Age, Gender, Race, Smoking, alcohol consumption, hypertension, hyperlipemia, diabetes. | | | | | | |
